# Dr. Willey J. P. Kingsley

**Published:** 1912-03

**Authors:** 


DR. WILLEY J. P. KINGSLEY, of Rome, died Jan. 26, aged 82. He was a graduate of the N. Y. Medical College, 1855. In 1895, he was Mayor of Rome.

